# Association Between Severity of Maternal Anemia and Adverse Fetomaternal Outcomes at Hayatabad Medical Complex

**DOI:** 10.7759/cureus.100297

**Published:** 2025-12-28

**Authors:** Shazia Tabassum, Nasreen Kishwar, Zara Usman, Faryal Rehman, Laila Ishtiaq

**Affiliations:** 1 Department of Obstetrics and Gynaecology, Hayatabad Medical Complex, Peshawar, PAK; 2 Department of Obstetrics and Gynaecology, Khyber Girls Medical College, Peshawar, PAK

**Keywords:** fetomaternal outcomes, low birth weight, maternal anemia, pakistan, preeclampsia, pregnancy complications

## Abstract

Introduction

Maternal anemia is still a major global health issue, especially in underdeveloped nations where its high incidence is a result of inadequate prenatal care and nutritional inadequacies. It has a negative impact on the health of both the mother and the fetus, increasing morbidity and mortality.

Objective

To determine the association between the severity of maternal anemia and adverse fetomaternal outcomes among pregnant women at Hayatabad Medical Complex, Peshawar.

Methodology

This cross-sectional study was carried out from January to December 2024 at the Hayatabad Medical Complex's Department of Obstetrics and Gynecology in Peshawar. In the study, 210 pregnant women were included through the use of consecutive sampling. The severity of anemia was categorized using WHO standards. The analysis was conducted using IBM SPSS Statistics version 26 for maternal and fetal outcomes data. To account for confounders, multivariate logistic regression was used, while chi-square and Fisher's exact tests were used for categorical variables. The p-value was deemed statistically significant if it was less than 0.05.

Results

Anemia was observed in 192 (91.4%) women, with 72 (34.3%) having mild, 90 (42.9%) moderate and 30 (14.3%) severe anemia. Maternal complications, including preeclampsia (p=0.004), preterm labor (p=0.012), and postpartum hemorrhage (p=0.018), were significantly associated with anemia severity. Fetal complications, including low birth weight (p<0.001) and neonatal intensive care unit (NICU) admission (p=0.006), were more frequent among women with severe anemia. After multivariate adjustment, severe anemia remained an independent predictor of adverse outcomes (adjusted odds ratio (AOR)=3.72, 95% confidence interval (CI): 1.68-8.26, p=0.001).

Conclusion

Adverse fetomaternal outcomes are highly correlated with the severity of maternal anemia. To avoid complications from anemia during pregnancy, early screening and prompt treatments are crucial.

## Introduction

Anemia during pregnancy remains a major public health challenge globally, particularly in low- and middle-income countries [1]. It is defined by the World Health Organization (WHO) as hemoglobin levels below 11 g/dL and classified as mild (10-10.9 g/dL), moderate (7-9.9 g/dL), or severe (<7 g/dL) [2]. Despite improvements in maternal healthcare, its prevalence continues to be high in South Asia due to nutritional deficiencies, poor socioeconomic status, and inadequate antenatal care [3]. Globally, nearly 40% of pregnant women are anemic, with over half of these cases occurring in developing nations [4].

Globally, anemia affects an estimated 36.8% of pregnant women, constituting a major public health concern worldwide [5]. According to the World Health Organization (WHO), approximately 35.5% of pregnant women aged 15-49 years are anemic [6]. The burden is particularly pronounced in South Asia, where prevalence rates may reach around 52%, substantially contributing to adverse maternal and neonatal outcomes [7]. In Pakistan, a recent meta-analysis reported a pooled prevalence of 54.9% among pregnant women [8], while comparable high prevalence has also been documented in other low-resource settings, such as Somaliland (50.6%) [9].

In Pakistan, maternal anemia remains a leading cause of maternal and perinatal morbidity and mortality [10]. Nearly half of all pregnant women in the country experience some degree of anemia, primarily due to iron deficiency, followed by deficiencies in folate and vitamin B12 [10-12]. Contributing factors include poverty, inadequate diet, repeated pregnancies, and limited awareness regarding nutritional supplementation [9,13,14]. Cultural food restrictions and insufficient access to healthcare further exacerbate the problem. The condition adversely affects maternal well-being, productivity, and fetal growth and survival [1]. 

The severity of maternal anemia has been shown to correlate directly with adverse pregnancy outcomes [11]. Women with moderate to severe anemia are at increased risk of preterm labor, preeclampsia, postpartum hemorrhage, and maternal mortality [11]. Fetal complications include intrauterine growth restriction, low birth weight, preterm birth, and stillbirth due to impaired oxygen delivery and placental insufficiency [12]. These adverse outcomes contribute substantially to perinatal morbidity and mortality in resource-limited settings [15]. 

Although several regional studies have explored this association, variations in socioeconomic conditions and healthcare access have led to inconsistent findings [4,7,8,14,15]. Evidence from tertiary care centers in Khyber Pakhtunkhwa remains limited, particularly regarding the relationship between anemia severity and fetomaternal outcomes [14]. 

Despite ongoing public health interventions, the burden of maternal anemia and its associated complications remains underexplored in this region [1,3,5-10]. Evaluating anemia based on severity rather than simple presence or absence provides important clinical nuance, as increasing severity of anemia has been shown to correspond with escalating risks of maternal and neonatal morbidity. Therefore, the primary objective of this study was to evaluate the association between the severity of maternal anemia, classified according to WHO criteria [2], and adverse maternal outcomes (including preeclampsia, postpartum hemorrhage, and preterm labor) as well as fetal outcomes (including low birth weight, intrauterine growth restriction (IUGR), stillbirth, and neonatal intensive care unit admission) among pregnant women delivering at a tertiary care hospital in Peshawar.

## Materials and methods

Study design, setting, and population 

A cross-sectional analytical study was conducted at the Department of Obstetrics and Gynecology, Hayatabad Medical Complex (HMC), Peshawar, Pakistan, between January 2024 and December 2024. HMC is a tertiary care teaching hospital providing comprehensive obstetric and gynecological services, including prenatal, intrapartum, and postnatal care, and serves both rural and urban populations of Khyber Pakhtunkhwa (KPK). During the study period, a total of 230 pregnant women were screened for eligibility. After applying the inclusion and exclusion criteria and ensuring complete data for all variables, 210 participants were consecutively enrolled. Each participant underwent assessment for anemia, and maternal and fetal outcomes were monitored throughout the delivery process.

Eligibility criteria and sample size

Pregnant women between the ages of 18 and 40 who gave their informed consent and gave birth at HMC throughout the study period were included in the study. The study excluded patients with multiple pregnancies, those who had received blood transfusions before admission, women with pre-existing hematological disorders like sickle cell anemia or thalassemia, and those with chronic medical conditions like renal failure, autoimmune diseases, or chronic infections. A sample size of 210 pregnant women was determined using the WHO sample size calculator. The calculation was based on the formula [16]

$n = \frac{Z^2 \, p(1 - p)}{d^2}$

assuming a 50% prevalence of maternal anemia to ensure maximum representativeness, a 95% confidence level (Z=1.96), and a 7% margin of error (d=0.07). This assumption was supported by a meta-analysis from Pakistan reporting a 54.9% prevalence of anemia among pregnant women [8] and a related study from Somaliland showing a 50.6% prevalence [9]. The estimated sample size was 196; however, in order to account for potential data loss or incomplete records, it was raised to 210. Until the necessary sample size was reached, all eligible women who met the inclusion criteria were enrolled using a sequential sampling procedure.

Data collection procedure

A standardized proforma, specifically designed for this study, was used to collect all relevant data (see Appendix 1). After obtaining written informed consent, participants underwent a comprehensive clinical assessment, including obstetric history, dietary habits, and socioeconomic background. Venous blood samples were collected on admission for hemoglobin estimation using an automated hematology analyzer (Sysmex XN-1000 Automated Hematology Analyzer; Sysmex Corporation, Kobe, Japan). Hemoglobin measurements were performed once for each participant, following manufacturer-recommended calibration and quality control procedures. All clinical staff involved in data collection were trained to ensure consistency, and standardized procedures were used to assess maternal outcomes, including postpartum hemorrhage (PPH), preeclampsia, and IUGR.

Operational definitions

Anemia was defined according to WHO criteria [2]. Participants with hemoglobin levels between 10.0 and 10.9 g/dL were classified as having mild anemia, those with hemoglobin between 7.0 and 9.9 g/dL as moderate anemia, and hemoglobin levels below 7.0 g/dL as severe anemia. Preterm birth was defined as delivery before 37 completed weeks of gestation, and IUGR was defined as birth weight below the 10th percentile for gestational age according to local reference charts.

Socioeconomic status was assessed based on monthly household income in Pakistani Rupees (PKR). Participants were classified as low (<25,000 PKR), middle (25,000-50,000 PKR), or high (>50,000 PKR) socioeconomic groups. This classification was used in the analysis to examine associations with maternal and fetal outcomes.

Every participant was monitored throughout labor and delivery. Maternal outcomes documented included maternal mortality, preterm labor, PPH, preeclampsia, and method of delivery. Fetal outcomes recorded included birth weight, Apgar score, preterm birth, IUGR, stillbirth, and admission to the neonatal intensive care unit (NICU). NICU admissions were primarily for low birth weight, preterm birth, IUGR, low Apgar scores, or other neonatal complications requiring specialized monitoring and care. Since NICU admission can result from multiple factors, it cannot be attributed solely to maternal anemia. Only participants with complete data for all relevant variables were included in the analysis.

Data analysis

Data were analyzed using IBM SPSS Statistics for Windows, Version 26.0 (IBM Corp, Armonk, NY, USA). Only participants with complete data for all relevant variables were included in the analysis. Clinical and demographic characteristics were summarized using descriptive statistics. Categorical variables, such as the degree of anemia, mode of delivery, and adverse outcomes, were presented as frequencies and percentages, while continuous variables, including age, hemoglobin level, and birth weight, were reported as mean±standard deviation (SD).

The Chi-square test or Fisher’s exact test, as appropriate, was used to evaluate associations between maternal anemia severity and fetomaternal outcomes. Multivariate logistic regression analysis was performed to identify independent predictors of adverse fetomaternal outcomes, adjusting for potential confounders including maternal age, parity, socioeconomic status, and interpregnancy interval for multigravida women. Prior to regression analysis, multicollinearity between covariates was assessed using variance inflation factor (VIF) statistics, and variables with VIF>5 were excluded. Variable selection for the final model was based on both clinical relevance and statistical significance (p<0.05). Statistical significance was set at a p-value of less than 0.05. All analyses were conducted on complete-case data to ensure the validity and reliability of findings.

Ethical considerations

The study protocol was reviewed and approved by the Institutional Ethical Review Committee of HMC, Peshawar (Approval No. 816/CD/HMC/2023; dated December 22, 2023). Written informed consent was obtained from all participants prior to data collection. All study procedures were conducted in accordance with the Declaration of Helsinki. Participant confidentiality was strictly maintained, and only anonymized data were used for research purposes.

## Results

**Figure 1:**
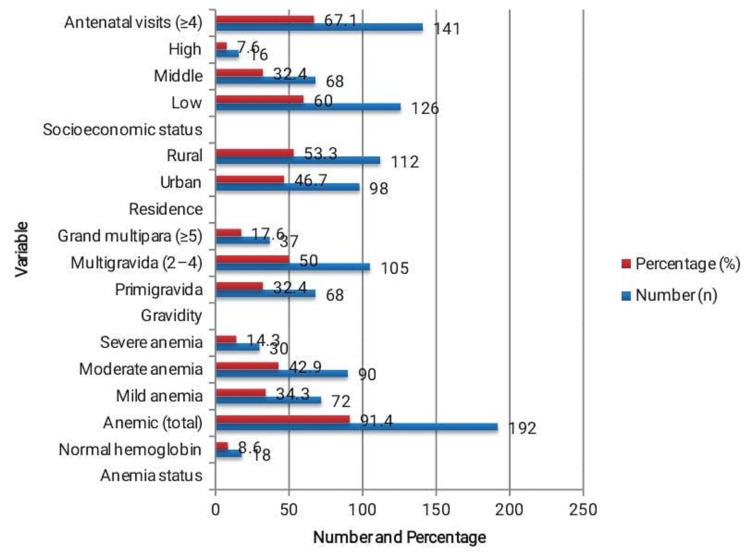
Demographic and Clinical Characteristics of the Study Population (n=210). The table presents the demographic and clinical profile of the participants, showing that most were multigravida women from rural, low-income backgrounds, with the majority attending adequate antenatal care visits.

A total of 210 pregnant women with a mean gestational age of 37.9±2.1 weeks and a mean age of 27.8±5.3 years were included in the study. Overall, 91.4% of mothers had anemia, whereas 8.6% had normal hemoglobin levels, as seen in Figure 1. Among the anemic women, moderate anemia was most common in 90 (42.9%), followed by mild anemia in 72 (34.3%) and severe anemia in 30 patients (14.3%). Most participants (105 (50.0%)) were multigravida, resided in rural areas (112 (53.3%)), and belonged to the low socioeconomic group (126 (60.0%)). Additionally, 141 (67.1%) women had four or more antenatal visits, indicating satisfactory healthcare utilization among the study population.

Table 1 summarizes the severity distribution of anemia among the 210 pregnant women. The average hemoglobin level was 9.4±1.8 g/dL, and 192 (91.4%) of the women were considered anemic. Among the subjects, 90 (42.9%) had moderate anemia, 72 (34.3%) had mild anemia, and 30 (14.3%) had severe anemia. Hemoglobin levels were normal in just 18 (8.6%) of the women. These results, which are in line with other area reports, show a substantial burden of anemia among pregnant women who visit HMC.

**Table 1:** Severity of Maternal Anemia among Participants The table illustrates the severity of maternal anemia, showing that moderate anemia was most common (42.9%), highlighting a significant public health concern.

Anemia Severity (WHO Classification)	Hemoglobin (g/dL)	Reference Range (g/dL)	Frequency, n (%)
Normal	≥11.0	12.0-16.0	18 (8.6)
Mild	10.0-10.9	12.0-16.0	72 (34.3)
Moderate	7.0–9.9	12.0-16.0	90 (42.9)
Severe	<7.0	12.0-16.0	30 (14.3)
Total	-	-	210 (100)

**Figure 2:**
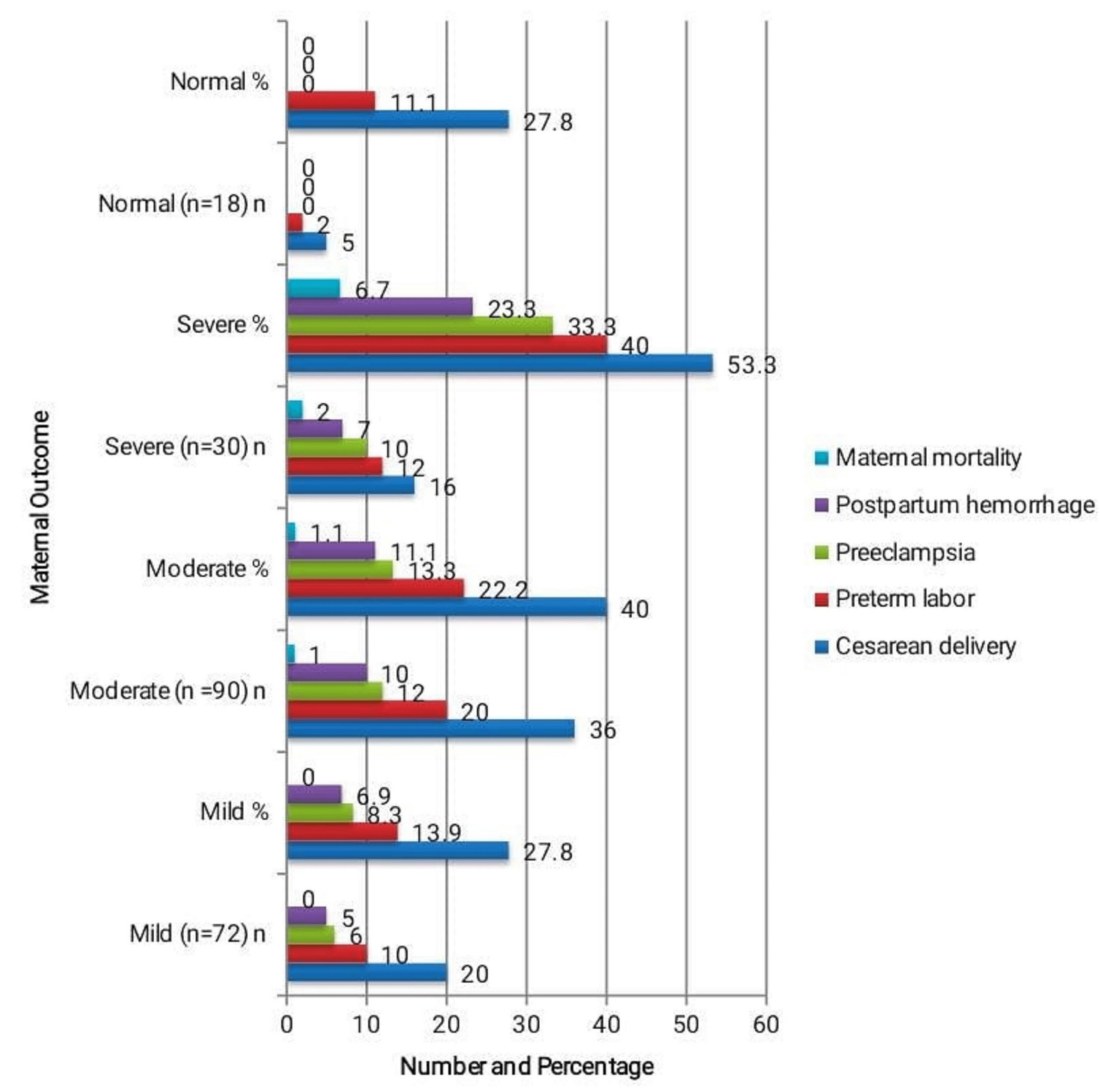
Association Between Severity of Maternal Anemia and Maternal Outcomes (n = 210). The relationship between maternal outcomes and the severity of anemia is displayed in Table 3, where moderate and severe anemia have noticeably greater rates of complications. Statistical analysis using chi-square tests (cesarean delivery: χ²=8.27, p=0.041; preterm labor: χ²=10.89, p=0.012; postpartum hemorrhage: χ²=9.95, p=0.018) and Fisher’s exact tests (preeclampsia: 10.66, p=0.004; maternal mortality: 7.83, p=0.032) confirmed that all differences were significant statistically (p<0.05).

The relationship between maternal outcomes and the severity of anemia in 210 individuals is depicted in Figure 2. Women with severe anemia had the highest rates of cesarean delivery, preterm labor, preeclampsia, and postpartum hemorrhage (16 (53.3%), 12 (40.0%), 10 (33.3%), and seven (23.3%)), respectively while only the severe anemia group experienced maternal mortality (2 (6.7%)). Chi-square tests showed significant associations between anemia severity and cesarean delivery (χ²=8.27, p=0.041), preterm labor (χ²=10.89, p=0.012), and postpartum hemorrhage (χ²=9.95, p=0.018), whereas Fisher’s exact tests indicated significant associations for preeclampsia (p=0.004) and maternal mortality (p=0.032). These findings demonstrate that increasing anemia severity significantly elevates the risk of maternal morbidity and mortality.

Fetal outcomes among 210 participants are shown in Table 2 according to the severity of maternal anemia. Compared to one (5.6%) neonate of a mother in the normal group, 19 (63.3%) neonates of participants with severe anemia had low birth weights. Preterm birth, IUGR, and NICU admissions were also more frequent with increasing anemia severity, occurring in 12 (40.0%), 10 (33.3%), and 11 (36.7%) neonates in the severe anemia group, respectively. The stillbirth rate was three (10.0%) in severely anemic mothers. Chi-square and Fisher’s exact tests confirmed significant associations for all fetal outcomes (low birth weight: χ²=27.21, p<0.001; preterm birth: χ²=11.63, p=0.009; IUGR: χ²=10.63, p=0.014; NICU admission: χ²=12.59, p=0.006; stillbirth: Fisher’s exact=7.92, p=0.048).

**Table 2:** Association between Severity of Maternal Anemia and Fetal Outcomes (n = 210). This table shows that negative fetal outcomes, such as low birth weight, preterm birth, intrauterine growth restriction (IUGR), neonatal intensive care unit (NICU) admission, and stillbirth, are substantially correlated with increasing maternal anemia severity. Statistical significance was confirmed using chi-square tests (low birth weight: χ²=27.21, p<0.001; preterm birth: χ²=11.63, p=0.009; IUGR: χ²=10.63, p=0.014; NICU admission: χ²=12.59, p=0.006) and Fisher’s exact test for stillbirth (7.92, p=0.048).

Fetal Outcome	Mild (n=72)	Moderate (n=90)	Severe (n=30)	Normal (n=18)	χ² / Fisher’s Exact Test (df)	p-value
Low birth weight (<2.5 kg)	15 (20.8%)	32 (35.6%)	19 (63.3%)	1 (5.6%)	χ²=27.21 (3)	<0.001*
Preterm birth (<37 weeks)	10 (13.9%)	22 (24.4%)	12 (40.0%)	2 (11.1%)	χ²=11.63 (3)	0.009*
Stillbirth	1 (1.4%)	3 (3.3%)	3 (10.0%)	0 (0.0%)	Fisher’s exact=7.92	0.048*
IUGR	9 (12.5%)	20 (22.2%)	10 (33.3%)	1 (5.6%)	χ²=10.63 (3)	0.014*
NICU admission	7 (9.7%)	18 (20.0%)	11 (36.7%)	1 (5.6%)	χ²=12.59 (3)	0.006*

After controlling for maternal age, parity, and socioeconomic level, Table 3 shows the results of a multivariate logistic regression analysis for predictors of unfavorable fetomaternal outcomes among 210 individuals. With an adjusted odds ratio (AOR) of 3.72 (95% confidence interval (CI): 1.68-8.26; Wald χ²=10.56, p=0.001), severe anemia continued to be a powerful independent predictor, and moderate anemia was also substantially linked to higher risk (AOR=2.14, 95% CI: 1.10-4.16; Wald χ²=5.09, p=0.024). Low socioeconomic status showed a borderline association (AOR=1.85, 95% CI: 0.98-3.47; Wald χ²=3.56, p=0.059), whereas maternal age >30 years (Wald χ²=1.35, p=0.245) and multiparity ≥3 (Wald χ²=0.77, p=0.383) were not significant predictors. These findings confirm that increasing anemia severity is an independent determinant of poor maternal and neonatal outcomes.

**Table 3:** Multivariate Logistic Regression Analysis for Predictors of Adverse Fetomaternal Outcomes (n=210). This table shows the multivariate logistic regression results for predictors of adverse fetomaternal outcomes. Wald χ² tests indicate that moderate anemia (Wald χ²=5.09, p=0.024) and severe anemia (Wald χ²=10.56, p=0.001) are independent and significant predictors, whereas low socioeconomic status showed a borderline effect (Wald χ²=3.56, p=0.059). Maternal age >30 years and multiparity ≥3 were not statistically significant.

Variable	Adjusted Odds Ratio (AOR)	95% Confidence Interval (CI)	Wald χ² (df)	p-value
Maternal age >30 years	1.43	0.78-2.61	1.35 (1)	0.245
Multiparity (≥3)	1.32	0.70-2.47	0.77 (1)	0.383
Low socioeconomic status	1.85	0.98-3.47	3.56 (1)	0.059
Moderate anemia	2.14	1.10-4.16	5.09 (1)	0.024*
Severe anemia	3.72	1.68-8.26	10.56 (1)	0.001*

These results demonstrate that increasing severity of maternal anemia is significantly associated with adverse maternal and neonatal outcomes. Severe anemia emerged as an independent predictor of poor fetomaternal outcomes, whereas maternal age >30 years and multiparity ≥3 were not statistically significant predictors. Low socioeconomic status showed a borderline association. NICU admissions, while more common among neonates of severely anemic mothers, may be influenced by multiple neonatal factors and cannot be solely attributed to maternal anemia.

## Discussion

Among pregnant women at the HMC in Peshawar, this study showed a substantial correlation between the degree of maternal anemia and unfavorable fetomaternal outcomes. The prevalence of anemia was high (91.4%), with moderate anemia being most common. Both maternal and fetal complications were notably higher in women with moderate to severe anemia. Even after adjustment for confounders, severe anemia remained an independent predictor of poor outcomes. These results demonstrate that maternal anemia, which has a direct impact on both infant survival and pregnancy safety, is a persistent public health issue in poor nations. 

The prevalence observed is consistent with reports from other low- and middle-income countries, where 45%-60% of pregnant women are anemic [17]. The predominance of moderate anemia mirrors regional patterns, likely due to nutritional deficiencies, poverty, and inadequate antenatal care [8]. Similar findings have been reported across South Asia and sub-Saharan Africa, emphasizing shared socioeconomic and dietary challenges [1]. 

Maternal complications such as preeclampsia, preterm labor, postpartum hemorrhage, and increased cesarean rates align with existing research from tertiary care hospitals across the region [18]. The physiological mechanism involves reduced oxygen-carrying capacity leading to placental hypoxia and endothelial dysfunction, predisposing anemic women to hypertensive and labor-related complications [19].

Fetal outcomes including low birth weight, intrauterine growth restriction, preterm birth, and increased NICU admissions-also paralleled global trends [19]. Severe anemia was strongly associated with low birth weight (63.3%) and stillbirths, similar to findings from other developing countries [5-7,15]. The relationship likely reflects chronic fetal hypoxia and inadequate nutrient transfer in anemic pregnancies [9,11].

Multivariate regression further confirmed anemia severity as an independent predictor of adverse outcomes, even after controlling for socioeconomic and obstetric factors [11]. This reinforces global evidence that maternal anemia is a key determinant of poor pregnancy outcomes regardless of parity or age [7].

Limitations and future suggestions

This study was conducted at a single tertiary care facility, which may limit the generalizability of the findings to other contexts. The cross-sectional design restricts causal inference, and temporal relationships between maternal anemia and adverse outcomes cannot be established. Certain potential confounding factors - including dietary intake, parasitic infections, adherence to iron and micronutrient supplementation, interpregnancy interval (IPI) among multigravida women, and factors contributing to NICU admission - were not fully evaluated. NICU admissions may result from multiple neonatal conditions and cannot be attributed solely to maternal anemia. Additionally, hospital-based recruitment may have introduced selection bias toward higher-risk pregnancies. Socioeconomic and educational factors, including illiteracy and lack of nutritional knowledge, likely contribute to the high prevalence of anemia and were not fully controlled in this study. Measurement variability and diagnostic criteria for maternal complications such as preeclampsia, PPH, and IUGR could also affect the results. Finally, handling of missing data was limited to inclusion of complete cases, which may affect external validity.

Future research should employ multicenter, longitudinal designs to establish causation and include a broader range of demographic and socioeconomic settings. Prenatal care programs should incorporate early screening, timely management of anemia, and educational interventions targeting nutritional awareness, particularly in low-literacy populations. Public health initiatives emphasizing dietary education, iron supplementation, and socioeconomic empowerment are essential to reduce the prevalence of maternal anemia. Biochemical studies investigating inflammation and iron metabolism could provide insights for targeted preventive strategies. Future studies should also account for multiple neonatal factors when evaluating outcomes such as NICU admission to improve causal inference and risk stratification.

## Conclusions

In this study, the severity of maternal anemia was strongly associated with adverse maternal and neonatal outcomes, including low birth weight, preterm labor, postpartum hemorrhage, and preeclampsia. Severe anemia remained independently associated with poor fetomaternal outcomes even after adjusting for maternal age, parity, and socioeconomic status. However, due to the cross-sectional design, causal relationships cannot be established. Unmeasured factors such as nutritional intake, adherence to iron supplementation, parasitic infections, chronic inflammatory conditions, and interpregnancy interval may have influenced the results. NICU admissions, although more frequent among neonates of severely anemic mothers, can result from multiple neonatal factors and cannot be solely attributed to maternal anemia. These findings underscore the importance of routine anemia screening, early diagnosis, and timely management during pregnancy, particularly in resource-limited settings, to reduce the risk of adverse maternal and neonatal outcomes.

## Appendices

Appendix 1

**Table 4:** Study Questionnaire / Data Collection Tool.

Section	Variable	Details / Categories	Data
Demographics	Participant ID	Unique identifier	
	Age (years)	Exact age	
	Residence	Rural / Urban	
	Socioeconomic status	Low (<25,000 PKR) / Middle (25,000–50,000 PKR) / High (>50,000 PKR)	
Obstetric History	Gravidity	Number of previous pregnancies	
	Parity	Number of live births	
	Interpregnancy interval (IPI)	Months between last delivery and current pregnancy	
	Number of antenatal visits	Exact number	
	History of pregnancy complications	Yes / No	
Clinical Assessment	Dietary habits	Assessed via questionnaire	
	Comorbidities	Chronic diseases (renal failure, autoimmune, etc.)	
	Blood transfusions before admission	Yes / No	
Laboratory	Hemoglobin (g/dL)	Measured on admission using Sysmex XN series	
	Anemia severity (WHO classification)	Mild (10.0–10.9 g/dL), Moderate (7.0–9.9 g/dL), Severe (<7.0 g/dL)	
Maternal Outcomes	Preterm labor	Yes / No	
	Postpartum hemorrhage (PPH)	Yes / No (WHO definition)	
	Preeclampsia	Yes / No (ACOG criteria)	
	Maternal mortality	Yes / No	
	Mode of delivery	Vaginal / Cesarean / Assisted	
Fetal Outcomes	Birth weight (grams)	-	
	Low birth weight (LBW)	<2500 g / ≥2500 g	
	Preterm birth	Delivery <37 weeks	
	Intrauterine growth restriction (IUGR)	Birth weight <10th percentile	
	Apgar score	1 min / 5 min	
	Stillbirth	Yes / No	
	NICU admission	Yes / No	
	Reason for NICU admission	LBW / Preterm / IUGR / Low Apgar / Other	
Other	Completeness of data	Complete / Incomplete	
